# Subannular left ventricular pseudoaneurysm following mitral valve replacement

**DOI:** 10.1186/1749-8090-3-28

**Published:** 2008-05-19

**Authors:** Narayanan Namboodiri, Santosh K Dora, Bejoy Thomas, Manoranjan Misra

**Affiliations:** 1 Department of Cardiology, Sree Chitra Tirunal Institute for Medical Sciences and Technology, Thiruvananthapuram, India; 2 Department of Imaging Sciences and Interventional Radiology, Sree Chitra Tirunal Institute for Medical Sciences and Technology, Thiruvananthapuram, India; 3 Department of Cardiothoracic Surgery, Sree Chitra Tirunal Institute for Medical Sciences and Technology, Thiruvananthapuram, India

## Abstract

Delayed development of left ventricular pseudoaneurysm is a rare late complication of mitral valve prosthesis and requires early surgical intervention. Here we describe the occurrence of such a complication diagnosed 6-months after the valve surgery in a 60-year-old lady. The anatomic delineation of subannular left ventricular pseudoaneurysm using multiple imaging modalities including CT angiography is also being discussed.

## Introduction

Delayed development of left ventricular pseudoaneurysm is a rare late complication of mitral valve prosthesis and requires early surgical intervention. Here we describe the occurrence of such a complication diagnosed 6-months after the valve surgery in an elderly lady. We discuss the anatomic delineation of subannular left ventricular pseudoaneurysm using multiple imaging modalities including CT angiography.

## Case history

**Figure 1:**
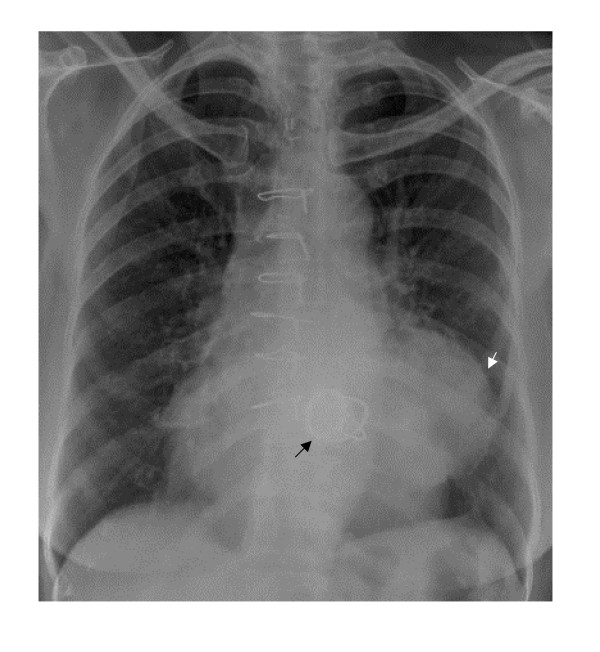
Chest radiograph in posteroaanterior view showed the rounded opacity along left cardiac silhouette.

**Figure 2:**
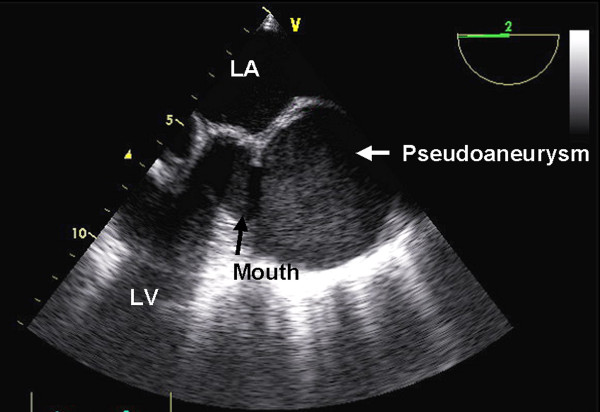
Transesophageal echocardiography in midesophageal short axis view showing the anatomical relations of pseudoaneurysm.

**Figure 3:**
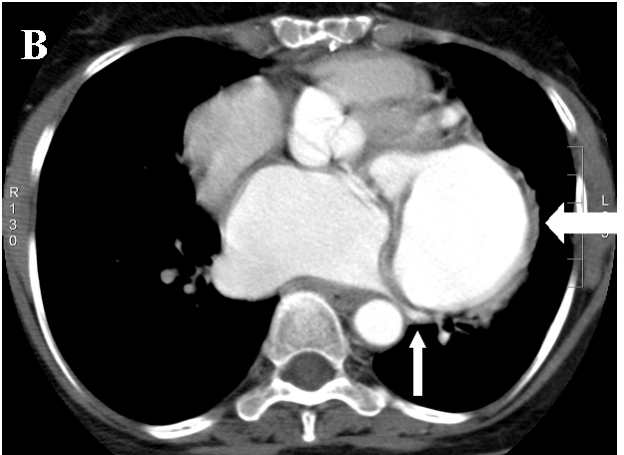
Axial source image of CT angiography showing the aneurysm (thick white arrow) compressing the left lower pulmonary vein (thin white arrow).

**Figure 4:**
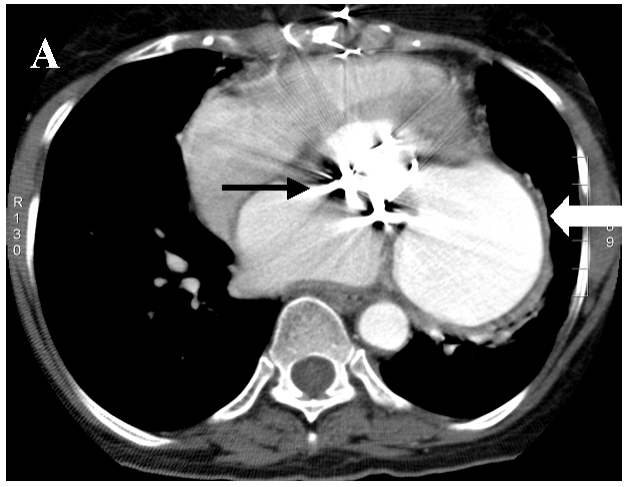
Axial source image of CT angiography showing the annular extension of the tear (thick white arrow -pseudoaneurysm, black arrow- valve).

**Figure 5:**
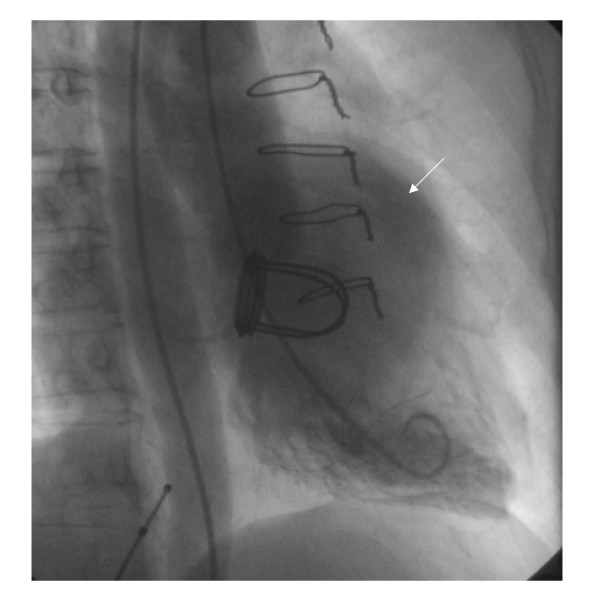
Coronary angiogram in left anterior oblique view showing displaced left circumflex artery with external compression (marked with white arrow) by the aneurysm.

**Figure 6:**
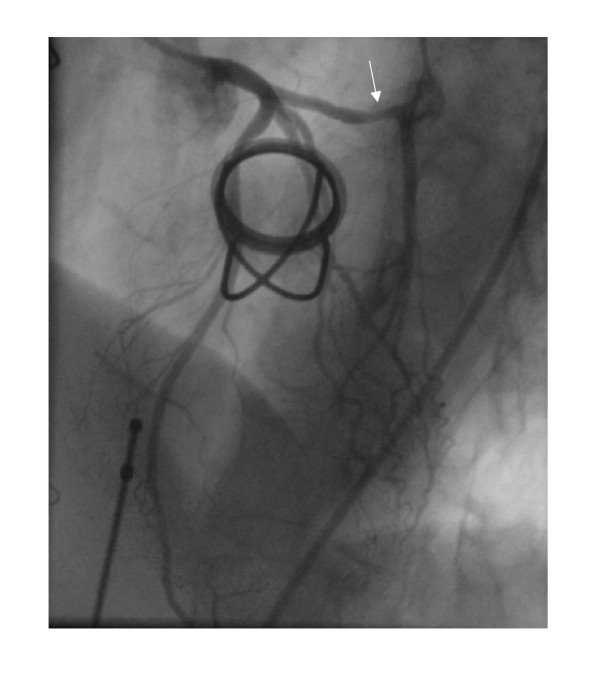
**Left ventricular angiogram in right anterior oblique view.** The large pseudoaneurysm is marked with white arrow.

A 60-year old lady underwent mitral valve replacement (MVR) with 28-Starr-Edward prosthesis in December 2005 for severe calcific mitral stenosis in our institute. She had also received saphaneous vein graft for a borderline lesion in mid left anterior descending artery (LAD) concomitantly. She was asymptomatic on follow up. Routine follow up transthoracic echocardiogram at 6-months postoperatively suggested a cavity at left atrioventricular groove communicating with the left ventricle (LV) at the posterobasal region of LV. Chest radiograph (posteroaanterior view) showed a rounded opacity silhouetting the left cardiac border (Figure 1). Transesophageal echocardiography (TEE) clearly delineated a 50 × 50 mm cavity filled with spontaneous echo contrast posterolateral to LV (Figure 2) beneath the left upper pulmonary vein. There was no turbulence demonstrable in the pulmonary veins. The aneurysm was communicating with left ventricular cavity through a 14 mm rent in the LV wall situated 7 mm away from the mitral annulus in horizontal and vertical midesophageal views. The walls of the cavity appeared to be formed only by pericardium suggesting a pseudoaneurysm of left ventricle. Mild paravalvular mitral regurgitation was also noted lateral to the prosthesis. No turbulence was noted in left pulmonary veins to suggest extraluminal compression. A computerized tomographic angiography (CTA) with 3-D reconstruction was done for better delineation prior to cardiac catheterization, which confirmed the site and size of the aneurysm. Left lower pulmonary vein (LLPV) (fig 3) and left lower bronchus showed extrinsic compression with no evidence of intraluminal obstruction. The rent which measured 20 mm in maximal diameter was in continuity with the mitral annulus in some planes (Figures 4). Coronary angiography showed occluded graft to LAD with TIMI 3 flow in native LAD. The left circumflex artery (LCx) was displaced posteriorly by the aneurysm with evidence of mild extrinsic compression (Figure 5). Subsequent left ventriculogram (Figure 6) showed dynamic filling of the pseudoaneurysm along the atrioventricular groove. Surgical repair of pseudoaneurysm through a left thoracotomy was planned. However, the patient was not willing for the surgical procedure.

## Comments

Left ventricular pseudoaneurysms are known to occur rarely as a delayed complication after MVR [1-3]. The cause of this complication is not clear, but its potential is present whenever there is early separation of the mitral annulus from the fibrous skeleton of the heart. Reoperation, endocarditis and oversizing have been reported to predispose to its formation. Many of the cases reported were asymptomatic as in our patient, but acute rupture is known to occur.

Proper anatomic delineation is essential in order to plan appropriate therapy. Unlike in the postmyocardial infarction cases, these aneurysms following MVR tend to be subannular in location [4]. The close proximity to the vascular structures and artifacts associated with the prosthesis make imaging of these aneurysms more difficult. Cardiac catheterization and echocardiography (transesophageal and transthoracic) have been used in the past with success [5-8]. However, complementing the existing imaging techniques with a new modality, CTA resulted in excellent delineation of anatomic details in this patient. TEE clearly demonstrated the communication to LV and the size of pseudoaneurysm. The echocardiographic features like the thickness of the cavity wall and narrow neck suggested it as pseudoaneurysm. TEE provided a full depiction of the LV and the adjoining pseudoaneurysm, enhancing the preoperative evaluation of the extent of the defect. However the relation to coronaries and bronchus was not clear on TEE. CTA well delineated the close proximity to LLPV and left lower bronchus. CTA thus provided detailed information about the extension of pseudoaneurysm. The detection of the continuity between the annulus and the rent as became evident on CTA precluded a percutaneous approach in the closure of pseudoaneurysm. Cardiac magnetic resonance imaging was not attempted due to the expected susceptibility artifacts due to the artificial valve.

Cardiac catheterization and coronary angiography could show the compression on LCx which was not clear either on TEE or CTA. However, no additional knowledge about other anatomic relations was obtained with this modality. Left ventricular angiogram confirmed the severity of paravalvular leak as mild only. Coronary angiography helped in ruling out the rare ischemic cause for pseudoaneurysm. TEE offered more reliable information about the site and size of pseudoaneurysm when compared to ventriculography. Comparing all modalities, CTA has a definite complementary role to TEE and left ventriculography in delineating the anatomical relations of LV pseudoaneurysm following MVR as a preoperative imaging modality.

Early surgical repair is recommended in these cases, particularly in large expanding pseudoaneurysms. To avoid the risk of repeat median sternotomy and adhesiolysis, left thoracotomy has been used in selected cases [1]. The surgical repair should be done on cardiopulmonary bypass (CPB) as attempts to suture the tear against pressure loaded, beating heart are likely to be unsuccessful. In case of suspected or detected thrombus in the aneurysmal cavity, dissection of the heart should initially be limited to the anterior surface, to enable placement of cannulas and institution of CPB. LV should be dissected free from the pericardium only after the aorta has been cross-clamped. The recommended surgical methods include internal, external or combined approaches [9,10]. Internal approach, the most preferred one in cases of rent involving the mitral annulus, posterior wall or large area of LV involves reopening the left atrium and the correction of the rent from within. Buttressed sutures are inserted from outside through the valve-sewing ring in lateral fashion avoiding LCx. In chronic cases, the neck of the pseudoaneurysm can be closed directly because of its firm fibrotic edges. Removal of the prosthesis has been suggested for better exposure in difficult cases. External repair by direct buttressed sutures is generally advocated for small defects limited to myocardium. In cases of widespread rents, combined approach removing the prosthetic valve and patching both the inside and outside of the ventricle might be recommended.

## Authors' contributions

NN carried out the echocardiographic study and prepared the manuscript. SKD participated in the coronary angiography and helped in the drafting of the manuscript. BT conducted the CT angiography and helped drafting the manuscript. MM helped in the surgical aspects of the manuscript. All authors read and approved the final manuscript.

## References

[B1] Ono M, Wolf RK (2002). Left ventricular pseudoaneurysm late after mitral valve replacement. Ann Thorac Surg.

[B2] Hirasawa Y, Miyauchi T, Sawamura T, Takiya H (2004). Giant left ventricular pseudoaneurysm after mitral valve replacement and myocardial infarction. Ann Thorac Surg.

[B3] Sanisoglu I, Duran C, Sagbas E, Akpinar B (2005). A left ventricular pseudoaneurysm due to mitral valve replacement. Eur J Cardiothorac Surg.

[B4] Yeo TC, Malouf JF, Oh JK, Seward JB (1998). Clinical profile and outcome in 52 patients with cardiac pseudoaneurysm. Ann Intern Med.

[B5] Carlson EB, Wolfe WG, Kisslo J (1985). Subvalvular left ventricular pseudoaneurysm after mitral valve replacement: two-dimensional echocardiographic findings. J Am Coll Cardiol.

[B6] Sakai K, Nakamura K, Ishizuka N, Nakagawa M, Hosoda S (1992). Echocardiographic findings and clinical features of left ventricular pseudoaneurysm after mitral valve replacement. Am Heart J.

[B7] Baker WB, Klein MS, Reardon MJ, Zoghbi WA (1993). Left ventricular pseudoaneurysm complicating mitral valve replacement: transesophageal echocardiographic diagnosis and impact on management. J Am Soc Echocardiogr.

[B8] Esakof DD, Vannan MA, Pandian NG, Cao QL, Schwartz SL, Bojar RM (1994). Visualization of left ventricular pseudoaneurysm with panoramic transesophageal echocardiography. J Am Soc Echocardiogr.

[B9] Prêtre R, Linka A, Jenni R, Turina MI (2000). Surgical treatment of acquired left ventricular pseudoaneurysms. Ann Thorac Surg.

[B10] Lanjewar C, Thakkar B, Kerkar P, Khandeparkar J (2007). Submitral left ventricular pseudoaneurysm after mitral valve replacement: early diagnosis and successful repair. Interact CardioVasc Thorac Surg.

